# Diversity of Color Vision: Not All Australian Marsupials Are Trichromatic

**DOI:** 10.1371/journal.pone.0014231

**Published:** 2010-12-06

**Authors:** Wiebke Ebeling, Riccardo C. Natoli, Jan M. Hemmi

**Affiliations:** ARC Centre of Excellence in Vision Science and Centre for Visual Sciences, Research School of Biology, ANU College of Medicine, Biology and Environment, The Australian National University, Canberra, Australia; Lund University, Sweden

## Abstract

Color vision in marsupials has recently emerged as a particularly interesting case among mammals. It appears that there are both dichromats and trichromats among closely related species. In contrast to primates, marsupials seem to have evolved a different type of trichromacy that is not linked to the X-chromosome. Based on microspectrophotometry and retinal whole-mount immunohistochemistry, four trichromatic marsupial species have been described: quokka, quenda, honey possum, and fat-tailed dunnart. It has, however, been impossible to identify the photopigment of the third cone type, and genetically, all evidence so far suggests that all marsupials are dichromatic. The tammar wallaby is the only Australian marsupial to date for which there is no evidence of a third cone type. To clarify whether the wallaby is indeed a dichromat or trichromatic like other Australian marsupials, we analyzed the number of cone types in the “dichromatic” wallaby and the “trichromatic” dunnart. Employing identical immunohistochemical protocols, we confirmed that the wallaby has only two cone types, whereas 20–25% of cones remained unlabeled by S- and LM-opsin antibodies in the dunnart retina. In addition, we found no evidence to support the hypothesis that the rod photopigment (rod opsin) is expressed in cones which would have explained the absence of a third cone opsin gene. Our study is the first comprehensive and quantitative account of color vision in Australian marsupials where we now know that an unexpected diversity of different color vision systems appears to have evolved.

## Introduction

Eutherian mammals only retain three of the five ancestral vertebrate photopigment genes: RH1 (rod opsin), SWS1 (short wavelength sensitive UV-/S-cones), and M/LWS (middle-to-long wavelength sensitive LM-cones; for reviews see [1]–[3]). This basic gene arrangement suggests dichromacy in all eutherian mammals with monochromacy only occurring secondarily where expression of the S-opsin gene has ceased, e.g. in marine species (e.g. [4]–[6]). Primates were long thought to be the only exception to this rule: Old World and some New World monkeys evolved trichromacy independently based on a duplication of the M/LWS gene [7] or X-chromosomal polymorphisms [8] (for a review see [3]). A few nocturnal primates also lost a functional S-opsin gene (e.g. [9], [10]). It has become evident, however, that mammalian color vision is more diverse. Bats are dichromats by histology [11], [12] with likely sensitivity in the UV range [12], [13] and the potential for color vision under photopic conditions [12] while lacking behavioral color vision under scotopic or low mesopic conditions [14]. Some bat species, however, have reverted to secondary monochromacy [15], and it is still unclear whether the M/LWS gene duplication in a Megabat [13] implies trichromatic color vision.

In contrast to placental mammals which use an SWS1 opsin as their short wavelength sensitive opsin, monotremes (echidnas and platypus) retain the SWS2 pigment gene in combination with an M/LWS opsin [16], [17]. The ancestral dimensionality of marsupial color vision, however, is not yet clear. Until recently, marsupials have been assumed to be “dichromatic” like most eutherian mammals. Two different cone types, containing either a SWS1 or a M/LWS opsin, have been found in both the Australian tammar wallaby [18]–[20] and the American opossums [21]–[25] (for a review see [26]). Four species of Australian marsupials (fat-tailed dunnart, honey possum, quenda, and quokka), however, seem to have the potential for trichromacy. Microspectrophotometry (MSP) in these species uncovered three cone spectral sensitivities [27], [28], and an estimated 3% of cones remained unlabeled when sequentially treated with S- and LM-opsin antibodies in whole-mount immunohistochemistry [28], [29]. Behavioral experiments confirmed functional trichromatic color vision in the fat-tailed dunnart, *Sminthopsis crassicaudata* [30]. The third cone opsin gene has, however, not yet been identified, and genetically, the dunnart is dichromatic [31], as are all marsupials studied so far [20], [23], [31], [32]. The quokka is a close relative of the wallaby (family: *Macropodidae*), and the question arises how diverse color vision in marsupials actually is and whether the tammar wallaby, *Macropus eugenii*, has been accurately classified as a dichromat [18], [19]. In addition, there is a clear mismatch in the proportion of dunnart “M”-cones between results from MSP and immunohistochemistry: 33% (20/61) of all individual cell MSP measurements were obtained from middle-wavelength sensitive cones which are assumed to be “M”-cones [27]. In contrast, only 3% of all cones in immunohistochemistry were found to be unlabeled and therefore assumed to be “M”-cones [29]. Unless Arrese et al. [27] were able to distinguish between “M”-cones and “L”-cones for their recordings, the MSP sampling frequency suggests that the third cone type might be as abundant as “L”-cones. As previous studies investigating the dunnart and the wallaby employed very different methods, the present study was designed to generate comparative and conclusive quantitative immunohistochemical data on the number of cone types in the retinae of both marsupial species.

The MSP results from Arrese et al. [27] show a positive correlation between the spectral sensitivity of the unknown “M”-cone opsin and the rod opsin. In the four species investigated, all four peak measurements lie within 3 nm of each other, suggesting that “M”-cone and rod spectral sensitivities are “synchronized”, even in species with unusually high sensitivity peaks of the rods like 512 nm in the fat-tailed dunnart [27], [28]. Part of this study was therefore aimed at testing whether rod opsin was expressed in marsupial cones which could explain the lack of a third cone opsin gene in these species. The recent finding of a duplicated RH1 gene in the dunnart genome [32] fits well with this argument.

## Methods

### Animals

Tammar wallabies (*Macropus eugenii*) were obtained from a breeding colony where they lived in small social groups in outdoor enclosures. Husbandry conditions were approved by the Animal Experimentation Ethics Committee under protocol R.VS.05.07. Retinal tissue for immunohistochemistry was collected from one adult female (5 kg) and two adult males (6.5 kg each). For the molecular biology part of this study, retinal tissue was extracted from two adult females (6 kg). All animals were sedated by general inhalation anaesthesia with isoflorane (2-chloro-2-[difluoromethoxy]-1,1,1-trifluoro-ethane) and euthanised through an intravenous injection (1 ml per kg body weight) of Valabarb (300 mg/ml) with 2% lignocaine into the tail vein.

Three fat-tailed dunnarts (*Sminthopsis crassicaudata*) from a breeding colony at the University of Adelaide, South Australia, were used in this study: one ex-breeder female (weight approx. 15 g; right eye for immunohistochemistry, left eye for molecular biology), one adult male (weight approx. 15 g; right eye for molecular biology), and one immature male (weight approx. 10 g; immunohistochemistry). Procedures were approved by Licence to Import and Take no. 2009374 and ethics protocol R.VS.27.08. Animals were sedated by general inhalation anaesthesia (isoflurane) and euthanised by a 1 ml intraperitoneal injection of Valabarb (300 mg/ml with 2% lignocaine).

### Immunohistochemistry

Wallaby eyes were fixed by ocular injection and transcardiac perfusion with 4% paraformaldehyde in 0.1 M phosphate buffer (PBS) at pH 7.4 for 30 min. Eyes were then removed and immersion-fixed in 4% paraformaldehyde for approximately 4 h before being stored in PBS at 4°C for several days prior to dissecting and embedding. Dunnart eyes were removed from unperfused animals and immersion-fixed in 4% paraformaldehyde for approximately 24 h prior to dissecting and embedding. Fixation was enhanced by administering a small corneal cut.

All retinae were dissected from scleral tissue and flattened by radial cuts, and the vitreous was removed. Retinal samples of about 1 mm^2^ from dorsal, central, or ventral locations were used for vertical and horizontal semi-thin sections (0.5 or 1 µm), i.e. perpendicular and parallel to the retinal layers, respectively. Samples were dehydrated in a sequence of ethanol baths, and then transferred to acetone before embedding in Medcast resin (Ted Pella, Redding/California). Sections were cut using a diamond knife (Diatome, Biel, Bern/Switzerland) and an ultra-microtome (Reichert-Jung, Depew/New York) and mounted on gelatine-coated glass slides. For the analysis of retinal photoreceptor types, consecutive horizontal 0.5 µm sections were mounted alternately on two different slides (a total of four slides with eight sections each). For the analysis of rod opsin expression in cone photoreceptors, eight consecutive horizontal 0.5 µm sections were mounted on a single slide.

The resin was removed by immersing the slide in a 0.5 M alcoholic solution of sodium hydroxide (15–20 min) and rinsing in ethanol (5 min), followed by three washes in distilled water (10 min each). Sections were then pre-incubated in 1% Normal Goat Serum (NGS) in 0.01% TritonX-PBS (45 min). Sections on each slide were immunolabeled with either of two monoclonal mouse antibodies that were designed to label rod opsin [33], Rho1D4 (Millipore, Billerica/Massachusetts) or Rho4D2 (kindly provided by R. Molday, Vancouver/British Columbia), and double-labeled with either of two polyclonal rabbit antibodies JH455, to label S-cones (expressed from a SWS1 gene), or JH492, to label LM-cones (expressed from M/LWS gene [34]; both kindly provided by P. Ahnelt, Vienna/Austria, and U. Grünert, Sydney/Australia by donation from J. Nathans, Baltimore/Maryland). Consecutive sections on different slides were treated alternately with either JH455 or JH492, but always with the same rod opsin antibody. The cone membrane marker Peanut Agglutinin (PNA) was used in combination with either rod opsin antibody, Rho1D4 or Rho4D2, on eight continuous sections per sample. Antibodies were diluted in 1% NGS in TritonX-PBS at concentrations of 1∶500 for Rho1D4 and Rho4D2, 1∶10000 for JH455, 1∶5000 for JH492, and 1∶50 for PNA. Slides were incubated at room temperature overnight and then rinsed in TritonX-PBS (3×10 min). Labeling was visualized with a secondary antibody (diluted 1∶1000 in 1% NGS in TritonX-PBS) conjugated with Alexa Fluor 488 (Invitrogen, Carlsbad/California; goat α-mouse) for rod opsin, Alexa Fluor 594 (goat α-rabbit) for JH455 and JH492, or Streptavidin 594 for the biotinylated PNA (goat α-rabbit). The secondary antibody was applied for 2 h at room temperature and rinsed off with TritonX-PBS (3×10 min). Slides were light-protected during incubation, rinses, cover slipping (sterile glycerol), and storage.

Fluorescent signals were specific to the binding of primary and secondary antibodies as they were absent in control experiments where either the primary or secondary antibody was omitted.

### Image Analysis

Immunolabeled sections were imaged using the 40× water-immersion objective of the LSM 5 Pascal confocal microscope system with LASOS HeNe 543 nm and Argon 488 nm scanning lasers (Zeiss, Jena, Thüringen/Germany). Settings for laser transmission, signal averaging, pinhole size, optimal detector, and amplifier gains were kept constant during collection of all images of the same sample, and were generally similar across all samples. The confocal microscope was also used to confirm the presence of cone outer segments in Differential Interference Contrast transmitted light.

Images (1024×1024 pixels) were exported and assembled using Adobe Photoshop (CS3, San Jose/California). Individual sections were reconstructed by alignment of images of neighboring parts of the section. Consecutive sections of the same retinal location were then aligned to create a digital stack. Individual cells could therefore be traced through several consecutive sections that had undergone alternating antibody treatment. As measured in vertical sections, wallaby and dunnart cone outer segments are approximately 5 µm long. Individual cone labels should therefore be visible in about ten consecutive horizontal sections, corresponding to five sections of each immunohistochemical protocol. This redundancy in the analysis provided a high level of accuracy in determining whether individual cones were labeled with either of our antibodies.

Images in the re-aligned digital stack of sequential horizontal sections were used to determine if all cones labeled with either antibody JH455 or JH492 or if any cones remained unlabeled, providing evidence of the presence of a third cone opsin to which neither of our primary antibodies could bind. To avoid mistaking non-retinal fluorescent signals for labels, e.g. dirt particles, a cone was only counted as labeled if the antibody label was present in at least two sections. Cones for which it was simply not possible to find repeated labels, for instance because they only became visible at the end of the image stack, were excluded from the analysis. In the case of faint JH455 labeling but strong JH492 labeling of cone outer segments in the dunnart retina, cones were counted as belonging to the LM-type. Faint labeling by either antibody that was unmatched by strong labeling of the other antibody was never observed.

To determine the number of cone types in each sample, cone positions were first marked in a Photoshop project layer using distinct circular gaps left by the cones in the green Alexa 488 rod opsin signal. These positions were then verified to contain cones by identifying cone oil droplets (see also [19], [35]) and inner and outer segments as visible in the background staining of the red Alexa 594 signal. For each cone antibody, a Photoshop project layer was used to record the position of labeled cone outer segments. Finally, all three layers were compared to determine whether individual cones were labeled with both cone antibodies and whether any cones remained unlabeled. The number of sampled cones was calculated using a custom-written Matlab script (Mathworks, Natick/Massachusetts).

The antibody JH455 which labels S-cones resulted in a certain degree of background staining which allowed us to detect unlabeled structures such as cone oil droplets or outer segments. This background information was used to determine whether a lack of labeling was due to missing or damaged cone outer segments. The fluorescent signal strength was sufficient to distinguish background staining and true labeling.

To analyze the potential labeling of cone outer segments with a rod opsin antibody, all PNA-positive cone outer segments were marked in a Photoshop project layer using the red Streptavidin 594 signal. The positions of these labels were then compared against the green Alexa 488 rod opsin label in all eight sections of the digital stack. Potential co-localizations were marked in a separate Photoshop project layer and carefully examined in the respective images as well as the images of the previous and following section of the stack.

### Molecular Biology

Immediately following euthanasia, eyes were removed, and retinae were dissected on ice, and placed in TRIzol (Invitrogen, Carlsbad/California). RNA was extracted and purified using the RNAqueous-Micro kit (Ambion, Austin/Texas). Concentration and purity (absorbance ratio 260 nm/280 nm) of the RNA were assessed by a spectrophotometer (ND-1000, NanoDrop Technologies Inc, Wilmington/Delaware), and only samples of purity values 1.9–2.2 were used. Reverse transcription from RNA to cDNA was performed on 1 µg of RNA per sample using the Superscript III kit (Invitrogen, Carlsbad/California). After one hour at 50°C, the reaction was terminated (15 min at 70°C), and samples were stored at 4°C. To check for contamination of DNA, negative controls were included by omission of the reverse transcriptase.

A fragment of the rod opsin (rhodopsin) gene sequence (rat: NM_033441 [36]; dunnart: AY159786 [37]; opossum: AY313946 [38]) was amplified using forward 5′-CTT CCC CAT CAA CTT CCT C-3′ (oligo no. 98454-001, batch no. 7424-053) and reverse 5′-CCC AGT GGR TTC TTG CC-3′ (oligo no. 98454-002, batch no. 7424-054) primers (http://www.sigmaaldrich.com/life-science/custom-oligos.html; Sigma-Aldrich, St. Louis/Missouri), matching a highly conserved region of vertebrate rod opsin. As a control gene, glyceraldehyde 3-phosphate dehydrogenase (GAPDH; rat: NM_017008) was amplified using forward 5′-AAC TTT GGC ATT GTG GAA GG-3′ (oligo no. 96679-004, batch no. 7253-080) and reverse 5′-TGT TCC TAC CCC CAA TGT GT-3′ (oligo no. 96679-005, batch no. 7253-081) primers, designed in human and mouse. The expected amplicon sizes were 833 bp for rod opsin and 222 bp for GAPDH. Only PCR results using the Platinum Taq kit (Invitrogen, Carlsbad/California) are presented here. According to the instructions of the kit, reaction tubes were set up with 25 µl of mastermix, containing 1 µl of cDNA. No template controls were included by replacing cDNA with DNase free water. The PCR started with 5 min at 94°C, cycled through 30 s at 94°C, 60°C, and 72°C forty times, and finished with 5 min at 72°C. Samples were stored at 4°C prior to visualisation by electrophoresis in a 1% w/v agarose gel in Tris/Borate/EDTA (TBE) buffer. For visualisation of DNA bands under UV light, the fluorescent dye GelRed (Biotium, Hayward/California) was added (5 µl per 100 ml gel). Samples and 100 bp ladders (Promega Corporation, Madison/Wisconsin) were combined with loading dye (Promega Corporation, Madison/Wisconsin) in a ratio of 1∶4 and 1∶1, respectively. The electrophoresis ran at 120 V for 2 h and continued for another 90 min to stretch out the 700 – 900 bp region. Pictures were taken at both time points.

## Results

### Antibody Specificity

**Figure 1:**
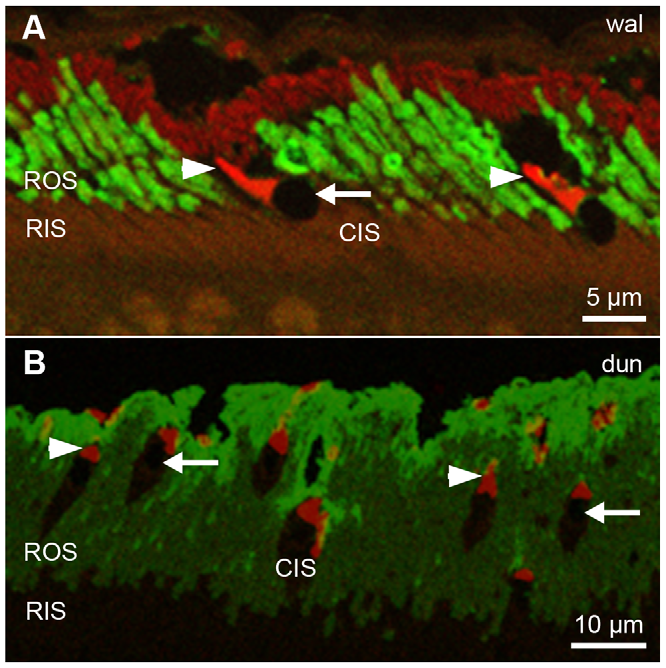
Photoreceptor outer segments immunolabeled with fluorescent secondary antibodies. Vertical cross sections (1 µm) of A) wallaby retina (“wal”) and B) dunnart retina (“dun”) treated with primary antibodies Rho1D4 (green fluorescent color tag, Alexa 488) and JH492 (red fluorescent color tag, Alexa 594) to visualize the rod opsin and LM-opsin photopigment, respectively. Immunolabeling was restricted to rod (ROS) and cone (arrowheads) outer segments; inner segments of both rods (RIS) and cones (CIS) remained unlabeled. Every cone contains an oil droplet (arrows).

Antibody labeling in vertical sections was clearly specific in both wallaby and dunnart (Figure 1). For all antibodies, labeling was restricted to the photoreceptor outer segments. Both cone and rod inner segments remained unlabeled. In both species, all cones were found to contain an oil droplet.

Retinal morphology is quite different in wallaby and dunnart tissue. Wallaby rod and cone inner segments lie at the same height in the tissue with the oil droplet distinctly marking the transition between cone inner and outer segment (Figure 1A). The dunnart photoreceptor layer, in contrast, is less regularly structured with cones being located at different heights within the long rod outer segments (Figure 1B). With regard to horizontal sections, this meant a lower degree of predictability of the position of cones and the transition between their outer and inner segments.

**Figure 2:**
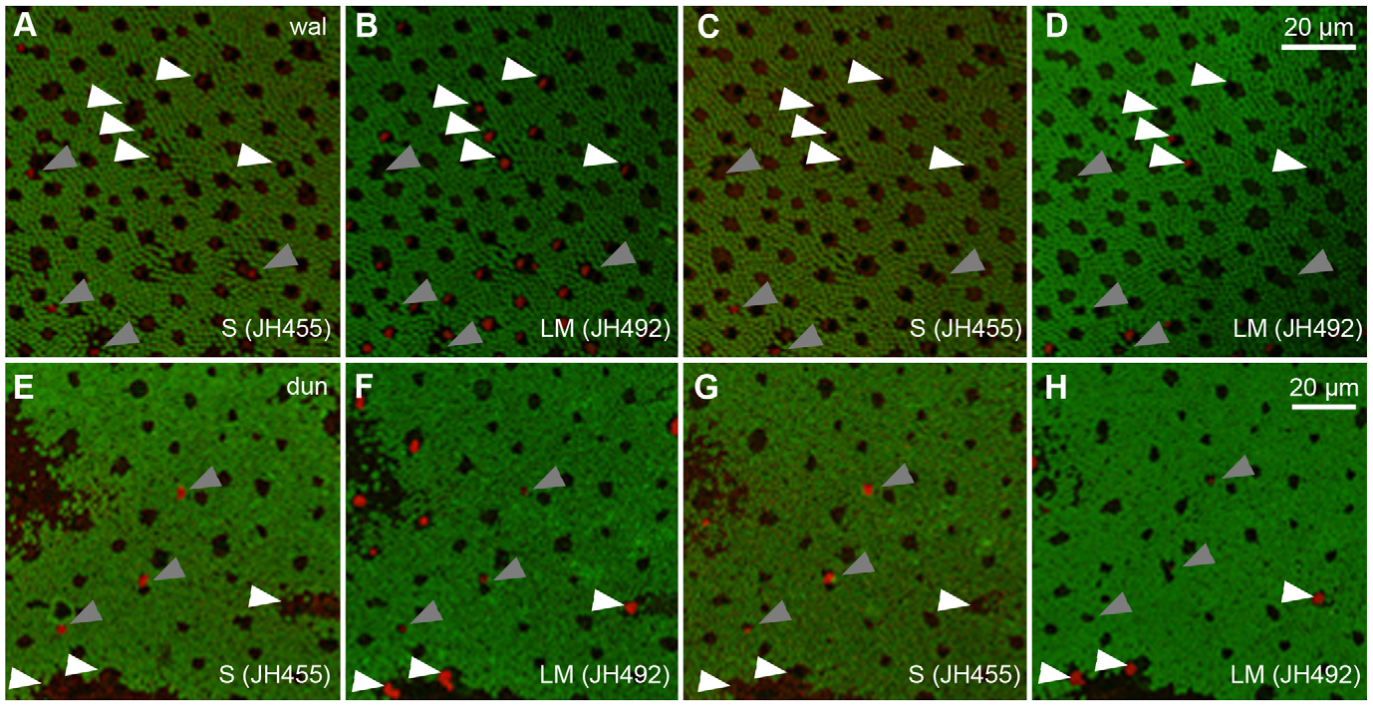
Alternating immunohistochemical protocols on sequences of consecutive retina sections. Four sections from a digital stack of A–D) wallaby (“wal”) and E–H) dunnart (“dun”) semi-thin (0.5 µm) horizontal retina sections immunolabeled with primary antibodies Rho1D4 (rod opsin, green) and A and C, E and G) JH455 (S-opsin, red) or B and D, F and H) JH492 (LM-opsin, red). Arrowheads mark the same selection of individual S-cones (gray) and LM-cones (white) in every section. A–D) All cones in this wallaby retina sample immunolabeled with either cone opsin antibody. E–H) Dunnart S-cones display a faint fluorescent label when treated with JH492. A substantial population (51%) of cones remained unlabeled by both opsin antibodies in this sample.

In the wallaby, cone opsin antibodies JH455 and JH492 selectively labeled distinct cone populations (Figure 2A–2D). In sequences of consecutive horizontal sections (0.5 µm), individual cones were either labeled by JH455 (specific to SWS1 or S-cones, gray arrowheads) or by JH492 (specific to M/LWS or M-cones, white arrowheads). We found no double-labeled cones. In the dunnart retina, the two antibodies also labeled distinct cone sub-populations (Figure 2E–2H). Most S-cones, however, also faintly labeled with JH492, even though JH492 produced almost no background staining.

### Number of Identifiable Cone Types

In both wallaby (Table 1) and dunnart (Table 2), we examined six retinal samples from both males and females across three retinal locations (dorsal, central, and ventral). From a total of 3206 wallaby cones, 88% were labeled by JH492 (LM-cones) and 11% by JH455 (S-cones). The S-cone percentage was as low as 4.6% in the ventral retina, increasing to 23% in the dorsal retina in accordance with results from Hemmi and Grünert [19]. In three of the six samples – comprising 70% of all analyzed cones – we found no unlabeled outer segments at all. In the remaining three samples we found a total of 27 potentially unlabeled cones.

**Table 1:** Distribution of cone types in six samples of tammar wallaby retina.

wallaby sample	n	LM labels	S labels	unlabeled
a♂ D	349	292/83.7%	50/14.3%	7/2.0%
b♂ D	566	430/76.0%	136/24.0%	0
a♂ DC	247	179/72.5%	56/22.7%	12/4.9%
♀ C	1040	969/93.2%	71/6.8%	0
a♂ V	358	333/93.0%	17/4.8%	8/2.2%
♀ V	646	616/95.4%	30/4.6%	0

Sample location D, dorsal; C, central; V, ventral; n, number of cones. Percentages are rounded to first decimal.

**Table 2:** Distribution of cone types in six samples of fat-tailed dunnart retina.

dunnart sample	n	LM labels	S labels	unlabeled
♀ D	624	394/63.1%	39/6.3%	191/30.6%
♂ D	222	163/73.4%	11/5.0%	48/21.6%
♀ C	1122	764/68.1%	74/6.6%	284/25.3%
♂ C	169	107/63.3%	6/3.6%	56/33.1%
♀ V	533	254/47.7%	42/7.9%	237/44.5%
♂ V	622	395/63.5%	52/8.4%	175/28.1%

Sample location D, dorsal; C, central; V, ventral; n, number of cones. Percentages are rounded to first decimal.

**Figure 3:**
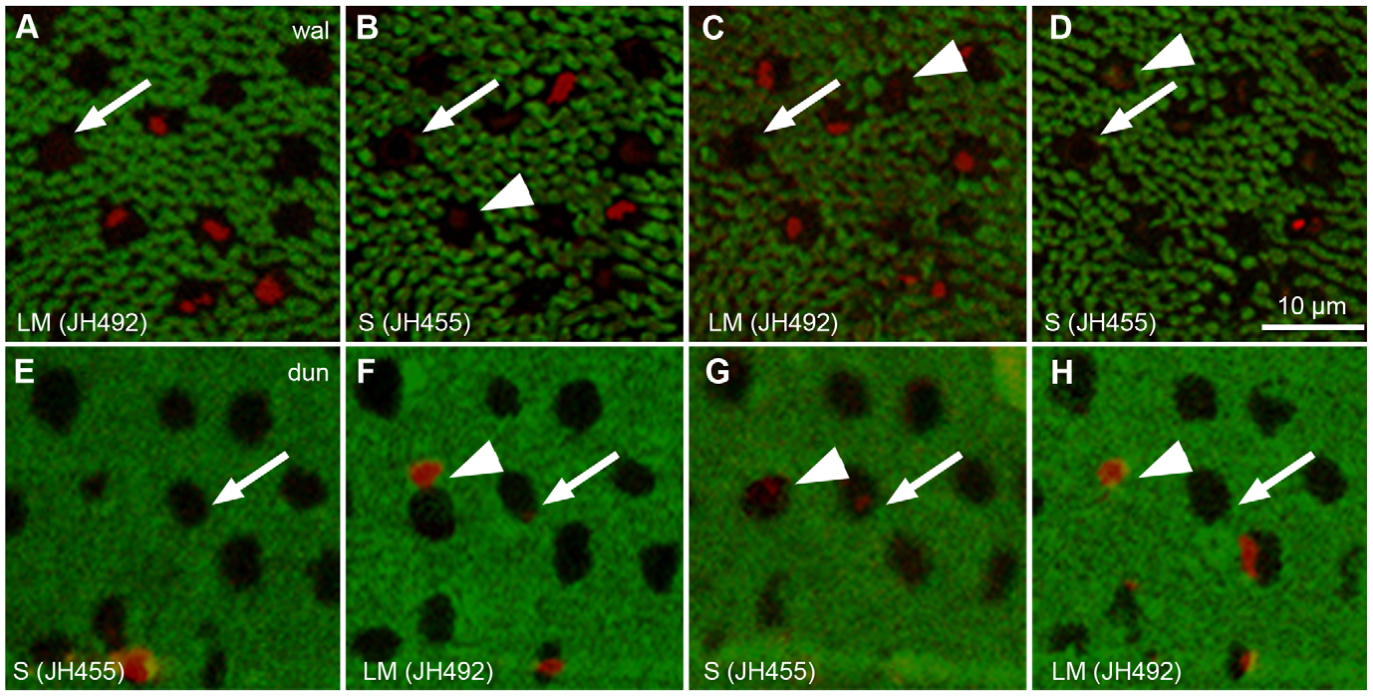
Outer segments of unlabeled cones in two immunohistochemical protocols. Four consecutive horizontal sections of A–D) wallaby (“wal”) and E–H) dunnart (“dun”) retina immunolabeled with primary antibodies Rho1D4 (rod opsin, green) and A and C, F and H) JH492 (LM-opsin, red) or B and D, E and G) JH455 (S-opsin, red). A–D) A wallaby cone remained unlabeled (arrow) by both cone opsin antibodies throughout the digital stack because its outer segment is missing. Neighboring cones are labeled by either antibody; their intact outer segments display in the background staining (arrowheads). E–H) The intact unlabeled outer segments of two cones in the dunnart retina (arrow and arrowhead in G) are visible in the background staining. Only one (arrowhead) labeled with a cone opsin antibody in F and H. The other cone (arrow) remained unlabeled in all sections.

To make sure we did not confuse local tissue damage, i.e. the absence of an outer segment, with the absence of labeling, we carefully examined cones in both species that remained unlabeled throughout the image stack in order to confirm the presence or absence of outer segment tissue in the section. In three of the potentially unlabeled cones, the image quality allowed us to confirm the absence of the outer segment (Figure 3A–3D). Neighboring cones clearly labeled with either of the two antibodies, and their intact outer segments were also visible as unlabeled structures (arrowheads in Figure 3B–3D) in consecutive sections. The fact that unlabeled cones were only found in some of the smaller samples strongly suggests that local tissue damage was responsible for the lack of labeling. In the wallaby retina, cone outer segments stick very tightly to the retinal pigment epithelium and frequently get damaged during dissection and/or embedding [19]. We therefore found no evidence of unlabeled cones in the wallaby retina.

In contrast to the wallaby, every sample of dunnart retina contained a substantial proportion of unlabeled cones (Table 2). JH492 labeled LM-cones also made up the largest proportion of cones in the dunnart retina (47.7%–73.4%), but unlabeled cones reached a relative percentage of 21.6% to 44.5%, clearly outnumbering the JH455 labeled S-cones (3.6%–8.4%).

Using the background staining in fluorescence images, the presence of intact outer segments of unlabeled cones could be confirmed in a minimum of 23.2% in sample ♀ V to a maximum of 95.8% in sample ♂ D of cones. An example is given in Figure 3E–3H where the background staining of the JH455 treated section (Figure 3G) displayed two unlabeled outer segments. One cone remained unlabeled in either immunohistochemical protocol (arrow) whereas the other one (arrowhead) was labeled in the preceding and following JH492 treated sections. Based on the two samples where the highest proportion of outer segments was visible in the fluorescent background staining (95.8% in ♂ D and 69.7% in ♂ V), we can confirm that the third cone type makes up at least 20% of all cones (20.7% and 19.6%, respectively). In the two samples where the fluorescent background staining provided the least amount of information, we took Differential Interference Contrast transmitted light microscope images after completion of the fluorescent image analysis to determine the presence or absence of outer segments. This additional analysis confirmed the presence of outer segments in 78.6% (♂ C) and 42.9% (♀ V) of unlabeled cones, resulting in a confirmed proportion of unlabeled cones of 26% and 23.5%, respectively. Unlabeled cones in the dunnart retina were, hence, not exclusively due to local tissue damage, and this immunohistochemical study can confirm the presence of three distinct cone types. The frequency of the third cone type lies somewhere between 20% and 25%.

### Does the Third Dunnart Cone Population express Rod Opsin?

**Figure 4:**
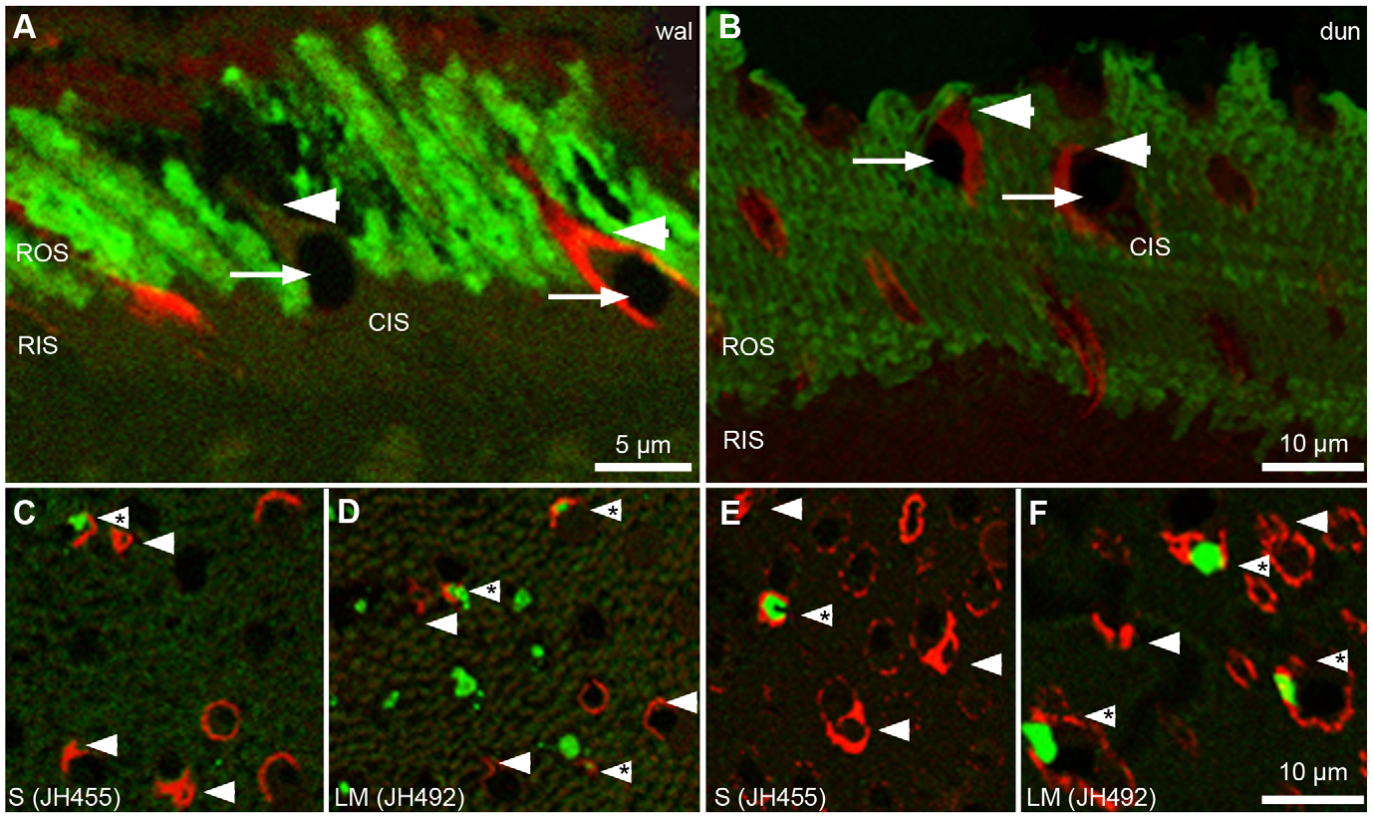
Labeling cone membranes with Peanut Agglutinin, PNA. A and B) Vertical cross sections (1 µm) of A) wallaby (“wal”) and B) dunnart (“dun”) retina treated with rod opsin primary antibody A) Rho1D4 or B) Rho4D2 (green fluorescence, Alexa 488) and the cone membrane marker PNA (red fluorescence, Streptavidin 594). Rod opsin labels are restricted to rod outer segments (ROS); rod inner segments (RIS) remain unlabeled. Cones contain oil droplets (arrow). A) PNA marks the outer membrane of some cones, not all (arrowhead) in the wallaby retina. B) All cones in the dunnart retina are PNA-positive but staining of outer (arrowhead) and inner segments (CIS) is patchy. C–F) Horizontal sections of C and D) wallaby and E and F) dunnart retina. PNA-positive cone outer segments (arrowheads) have an open chamber where the photopigment C and E) S-opsin and D and F) LM-opsin is located (asterisks).

We used rod opsin antibodies to test whether rod opsin was expressed in the third dunnart cone type. If this were true, the previous analysis suggests a proportion of 20–25% rod opsin-positive cones. Our immunohistochemical protocol was initially designed to detect very low numbers of the third cone type. We used two primary antibodies to detect different terminal regions of the rod opsin molecule, Rho1D4 (C-terminal) and Rho4D2 (N-terminal). Both antibodies were found to label rod outer segments only, leaving rod inner segments unlabeled (see vertical sections in Figure 4A,4B). The cone membrane marker Peanut Agglutinin (PNA) was used to visualize the location of the photopigment in cone outer segments. In the wallaby retina, only a subset of cones were found to be PNA-positive (33% in 726 cones sampled in a preliminary test) with labeling being either consistently strong or completely absent (see vertical section in Figure 4A). In the dunnart retina, PNA labeled all cones, but labeling strength along the membrane varied such that at times the PNA label was reduced to a spotted pattern (see vertical section in Figure 4B). In horizontal sections, the location of the photopigment was identified as an open chamber in the cone outer segment (arrowheads in Figure 4C–4F). Co-localization of rod opsin was therefore defined as a green rod opsin signal inside the open chamber of a PNA-labeled cone outer segment. In order to exclude confounding non-specific fluorescent signals such as those emitted by dirt particles on single sections, this co-localization had to occur in at least two consecutive sections (“repeated”). Control experiments using PNA with either JH492 (LM-opsin) or JH455 (S-opsin) in an immunohistochemical protocol showed that PNA labeled both S-cones and LM-cones (asterisks in Figure 4C,4D) and that PNA-negative cones also came from both cone populations (LM-cones in Figure 4D). Control experiments in the dunnart revealed that, firstly, PNA labels were not different between S-cones and LM-cones (Figure 4E,4F) and that the number of JH492-negative PNA-positive cone outer segments in the control experiment (arrowheads without asterisks in Figure 4F) was much higher than the proportion of S-cones (approximately 7%, see Table 2). Hence, at least a certain percentage of the third cone type had to be PNA-positive.

**Figure 5:**
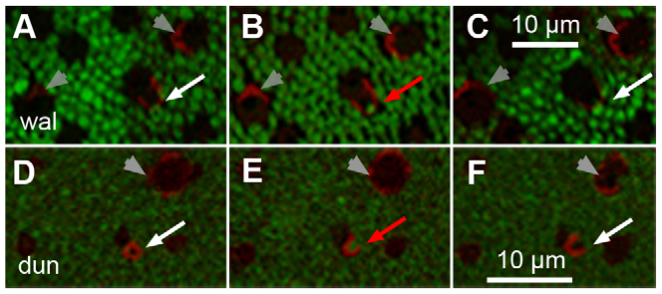
Singular co-localizations of rod opsin in a PNA-positive cone outer segment. Sequence of three consecutive horizontal sections of A–C) wallaby (“wal”) and D–F) dunnart (“dun”) retina treated with rod opsin antibody Rho1D4 (green) and PNA (red). Sections B and E) show the potential co-localization of the rod opsin label in the outer segment chamber of a cone as marked by PNA (red arrow). This co-localization was not observed in the previous A and D) or following C and F) section of the digital stack (white arrows). Neighboring PNA-positive cones are not rod opsin-labeled (arrowheads).

Eight wallaby (Table 3) and seven dunnart (Table 4) retina samples were analyzed, most of which had been treated with the rod opsin antibody Rho1D4 and some with Rho4D2. The image stacks of eight consecutive horizontal sections (0.5 µm) were examined for the repeated occurrence of co-localized rod opsin labels in PNA-positive cone outer segments. In neither species, however, was this ever observed. Three wallaby samples and six dunnart samples contained a total of twelve co-localized signals in a single section, but none of them was visible in the previous or following section of the image stack (Figure 5A–5F). Such singular co-localizations were found in both Rho1D4 and Rho4D2 treated sections (Tables 3,4). The expected proportion of 20–25% rod opsin labels clearly lay outside the 95% confidence interval of the observed eight singular co-localizations (CI from 0.47% to 1.80%).

**Table 3:** Occurrence of co-localized rod opsin labels in PNA-positive cone outer segments of tammar wallaby retina samples.

Wallaby sample	n	rod opsin antibody	singular co-localization	repeated co-localization
a♂ D	82	Rho1D4	0	0
b♂ D	244	Rho1D4	1	0
♀ D	137	Rho1D4	1	0
b♂ VC	102	Rho4D2	0	0
b♂ VC	45	Rho1D4	0	0
a♂ V	300	Rho1D4	2	0
b♂ V	25	Rho1D4	0	0
♀ V	78	Rho4D2	0	0
Total	1013		4	0

Sample location D, dorsal; C, central; V, ventral; n, number of PNA-labeled cone outer segments.

**Table 4:** Occurrence of co-localized rod opsin labels in PNA-positive cone outer segments of fat-tailed dunnart retina samples.

Dunnart sample	n	rod opsin antibody	singular co-localization	repeated co-localization
♀ D	56	Rho1D4	1	0
♂ D	128	Rho1D4	1	0
♀ C	187	Rho1D4	1	0
♂ C	21	Rho4D2	0	0
♀ V	113	Rho1D4	1	0
♀ V	156	Rho4D2	3	0
♂ V	210	Rho1D4	1	0
Total	871		8	0

Sample location D, dorsal; C, central; V, ventral; n, number of PNA-labeled cone outer segments.

**Figure 6:**
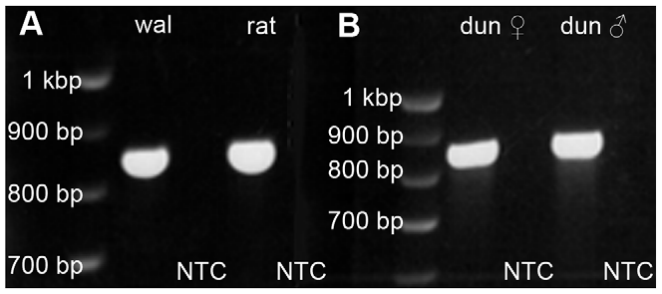
Molecular size of rod opsin in three mammalian species. The rod opsin gene sequence was isolated from retinal mRNA of A) rat and wallaby (“wal”) and B) dunnart (“dun”) by primers, reverse transcribed, amplified by PCR, and visualized by Agarose gel electrophoresis. A single band at approximately 850 bp molecular size displays in all species. Splice isoforms do not occur in the primed sequence of the rod opsin gene. Tissue from a female (♀) and a male (♂) dunnart was used, but delivered the same band. No template controls (NTC) were run for each species (black lanes).

In neither the wallaby nor the dunnart did we find any cones that were labeled by our rod opsin antibodies. In order to make sure that these cones do not express a different isoform of rod opsin that is not immunohistochemically compatible with our rod opsin antibodies, we screened retinal RNA of dunnart, wallaby (dichromatic marsupial control), and rat (dichromatic placental control) for the expression of multiple rod opsin splice isoforms. The omission of one of the five rod opsin gene exons by alternative splicing could account for the lack of immunoreactivity with both rod opsin antibodies. Primer binding sites were located in exons 1 and 5 of the rod opsin gene to investigate a 833 base pairs (bp) gene fragment that is highly conserved and therefore the most likely region to contain the isoforms. Our analysis was therefore sensitive to splice isoforms by omission of exons 2, 3, or 4 but would not have detected splice isoforms by omission of exons 1 or 5. Predicted isoforms of dunnart rod opsin would have consisted of 668 bp (exon 2 omitted), 669 bp (exon 3 omitted), or 595 bp (exon 4 omitted). Procedures of extraction, transcription, and amplification of retinal RNA were successful as confirmed by using GAPDH as a control gene (222 bp; data not shown). The experiment detected only a single rod opsin amplicon in all species tested (Figure 6). The banding pattern as visualized by agarose gel electrophoresis clearly showed a DNA product sized between 800 and 900 bp, even after maximum separation. Female and male dunnart tissue delivered identical results.

## Discussion

The data confirmed previous reports indicating that 1) the wallaby retina contains only two cone types and 2) a considerable proportion of cones in the dunnart retina remains unlabeled by S- and LM-opsin antibodies. Employing immunohistochemistry and molecular biology, we found no evidence of rod opsin being expressed in dunnart cones. The underlying photopigment of this third cone type is therefore still unknown.

### Dichromatic and Trichromatic Marsupials

Our carefully designed immunohistochemistry protocol provided no evidence of a third cone population in the wallaby retina, but confirmed the existence of three cone types in the retina of the fat-tailed dunnart. Between 20 and 25% of all dunnart cones remained unlabeled by both antibodies used which consistently detect S- and LM-cones in mammals [34], [39]–[41]. The finding of only two cone types in the tammar wallaby agrees with an earlier study which was based on a much smaller set of cones [19]. While it is difficult to prove the absence of a very small percentage of unlabeled cones, we analyzed 2252 cones across the three samples where all outer segments were intact, and we found no unlabeled cones. Including the 445 cones measured by Hemmi and Grünert [19], not a single unlabeled cone was found in 2697 cones. If there is a third cone type at all, it would have to be extremely sparse. Even if only half a percent of all wallaby cones contained a third cone opsin, we would have expected to find an average of about 13 cones in these samples. The wallaby, therefore, clearly has only two cone types, in agreement with the number of cone opsin genes [20] present in this species. The only other alternative explanation of how a third cone type could have remained undetected in this study would require these cones to double-label with either JH455 or JH492. Earlier behavioral work by Hemmi [18], indicating that wallabies do indeed have dichromatic color vision, supports the present data. In order to confirm this report, a more detailed behavioral study will be needed to address the dimensionality of wallaby color vision by ruling out the presence of a very small minority of cones of a third spectral type.

The present study supports the qualitative report by Arrese et al. [29] that suggested the presence of a third cone type in the dunnart retina. The frequency of unlabeled cones we found in the dunnart retina, however, was much higher (20–25%) than the previously estimated 3% [29]. Surprising as this difference in proportions is, our estimate now offers a much more realistic match to the frequency of “M”-cones identified by microspectrophotometry [27]. In their study, absorption spectra were obtained from 38 L-cones, 20 M-cones and 3 S-cones. These numbers suggest an M-cone ratio of about 33%. M-cones were clearly much more abundant than S-cones which based on Arrese et al. [29] make up approximately 7% of all cones. While sampling biases make estimating cell proportions from such MSP measurements unreliable, it is difficult to reconcile these numbers with a 3% relative frequency. It is possible that the consecutive application of antibodies to whole-mounts of dunnart retina in the study of Arrese et al. [29] caused the majority of unlabeled cones to label with JH492, leading to a strong overestimation of L-cones and underestimation of M-cones.

For the wallaby, our results agree well with Hemmi and Grünert's [19] findings, the only notable difference being the absence of double-labeling of S-cones by the LM-opsin antibody in the current study. Given the immunohistochemical protocols were almost identical, this difference is most likely due to our use of fluorescent secondary antibodies that provide almost binary signal amplification. This suggests that the putative double-label found by Hemmi and Grünert [19] was in fact non-specific labeling. Interestingly, however, we found that dunnart S-cones displayed a faint JH492 double-label which was not observed in whole-mounts [29]. Investigation of this potential co-expression will be a future avenue of exploration.

Given such a high percentage of M-cones, it will be interesting to investigate their exact distribution across the retina of the dunnart and the other trichromatic marsupial species and to determine how this cone type is integrated with the standard mammalian S- and L-cones.

### The Third Cone Opsin

The most intriguing aspect of marsupial trichromacy is the apparent lack of a third cone opsin gene. With peak absorption around 500 nm [27], [28], the third cone type is medium-wavelength sensitive, similar to ancestral opsins RH2 or SWS2, but neither gene is retained in the dunnart genome [32]. Melanopsin is a retinal pigment with peak absorption around 480 nm (e.g. [42]), and it was found to be expressed in a low percentage of cones in the peripheral human retina [43]. In the dunnart retina, however, melanopsin is restricted to the ganglion cell layer [44].

Based on the fact that all marsupial M-cones appear to have a peak spectral sensitivity of about 500 nm (for reviews see: [26], [45]), it is tempting to suggest that these cones contain an opsin expressed from the RH1 gene, which is usually expressed in rods only [45], [46]. Given the dunnart genome contains two copies of the RH1 gene [32], one copy could be expressed in rods and one in cones. Across the four marsupial species analyzed, there is an apparent positive correlation between the rod opsin peak absorbance and that of the third cone spectral sensitivity of each individual species [27], [28]. Our hypothesis of rod opsin being expressed in cones would explain this correlation and appears feasible in the light of the situation in amphibians and reptiles where the dichotomy of rods and cones is not evident at the level of photopigment expression (e.g. [47]-[51]). However, in our analysis of 871 dunnart cones we did not detect any clear labeling of the outer segment by either rod opsin antibody Rho1D4 or Rho4D2, as indicated by a fluorescent signal in more than one horizontal section. We also did not find any evidence of cone labeling with our rod opsin antibodies in the wallaby. The antibodies used should be able to bind to rod opsin if contained within the membrane disc, unlike for example Rho4A2 [52].

It is not clear though whether the absence of rod opsin antibody labeling really indicates the absence of rod opsin in cones. There are a number of reasons why our antibodies might not have recognized their target. While both antibodies clearly labeled rods in both dunnart and wallaby, the particular rod opsin epitopes might not be detectable or accessible. If the two RH1 gene copies in the dunnart genome [32] differ in exons 1 and/or 5, which have not been sequenced yet, antibody binding sites might be affected, and only the “rod-specific rod opsin” epitope may be compatible with our antibodies. Structurally different rod opsin epitopes can also be the result of alternative splicing which is more frequent in duplicated genes [53], making it more likely to occur in the two dunnart RH1 copies [32]. Retinal RNA is known to be alternatively spliced into G-protein isoforms with different C-termini [54], and alternative splicing also occurs in opsin-related genes in the retinal pigment epithelium and Müller cells [55]. We found, however, no evidence of alternative splicing in the dunnart. With the advent of High-Throughput Sequencing, it would be possible to investigate Single Nucleotide Polymorphisms that potentially change the amino acid sequence if they are non-synonymous. In the RH1 gene of several guppy strains, rod opsin sequences were found not to vary much, and only one SNP was non-synonymous [56].

### Marsupial Color Vision

The difference between the dichromatic wallaby and several other marsupials that were shown to have three different cone types presents an intriguing puzzle. If the ancestral marsupials were dichromatic like the wallaby and the American opossums [21]-[25] with two cone opsin genes (SWS1 and M/LWS), a third cone type must have evolved at least twice independently both in the “polyprotodonts” and in the diprotodonts, the latter being a monophyletic group to which the wallaby, the quokka, and the honey possum belong. A number of these apparently trichromatic species have UV-sensitive S-cones [23], [27], [28]. Improving color vision in the middle-to-long wavelength range could well have been the driving force to evolve a third spectral sensitivity. Placental mammals with UV-sensitive S-cones, such as rodents [57], [58], have been shown to perform poorly in color discriminations.

While particular attention has been paid to explain the re-evolution of trichromacy in Old and New World monkeys, for example by modeling whether dichromatic individuals would be disadvantaged in detecting fruits and flowers (e.g. [59], [60]), a similarly plausible “feeding hypothesis” does not exist for marsupials. There is no obvious reason why wallabies should be dichromatic but the related quokka trichromatic, a species with very similar habitat and lifestyle and peak spectral sensitivity of the S-cone in the violet-to-blue range [18], [20], [28]. In the potentially trichromatic honey possum, a first modeling study suggested that trichromacy is only beneficial in some aspects of the animal's visual ecology [61].

An alternative scenario is that only the early Australian marsupials, the common ancestors of the “polyprotodonts” and of the diprotodont lineage, but not the American opossums [62], were trichromatic. The wallaby and potentially other marsupial species would have selectively reduced spectral sensitivity to secondary dichromacy. Again, it is unclear, however, why this would occur in the wallaby, but not in the quokka.

Information about the color vision abilities of Australian marsupials is still extremely limited. We have now established that the differences between the retina of the dichromatic wallaby and the trichromatic dunnart are genuine and are not accounted for by methodological differences in analysis. They form the basis for their color vision system. As a next step, it would be particularly interesting to test other marsupials using both anatomical and behavioral experiments in order to reconstruct the evolution of color vision in mammals and to understand why such closely related species as the wallaby and the quokka seem to have evolved different color vision systems.
